# Biopolymeric Nanogel as a Drug Delivery System for Doxorubicin—Improved Drug Stability and Enhanced Antineoplastic Activity in Skin Cancer Cells

**DOI:** 10.3390/ph17020186

**Published:** 2024-01-31

**Authors:** Lyubomira Radeva, Maya M. Zaharieva, Ivanka Spassova, Daniela Kovacheva, Ivanka Pencheva-El Tibi, Hristo Najdenski, Krassimira Yoncheva

**Affiliations:** 1 Faculty of Pharmacy, Medical University of Sofia, 1000 Sofia, Bulgaria; l.radeva@pharmfac.mu-sofia.bg (L.R.); itibi@pharmfac.mu-sofia.bg (I.P.-E.T.); 2 The Stephan Angeloff Institute of Microbiology, Bulgarian Academy of Sciences, 1113 Sofia, Bulgaria; zaharieva26@yahoo.com (M.M.Z.); hnajdenski@gmail.com (H.N.); 3 Institute of General and Inorganic Chemistry, Bulgarian Academy of Sciences, 1113 Sofia, Bulgaria; ispasova@svr.igic.bas.bg (I.S.); didka@svr.igic.bas.bg (D.K.)

**Keywords:** doxorubicin, nanogel, drug stability, chitosan, albumin, skin cancer

## Abstract

In this study, doxorubicin was loaded in a chitosan–albumin nanogel with the aim of improving its stability and exploring the potential of the system in the treatment of skin cancer. Infrared spectroscopy and X-ray diffraction confirmed the encapsulation of the drug. Transmission electron microscopy revealed the spherical shape of the nanogel particles. The drug-loaded nanogel was characterized with a small diameter of 29 nm, narrow polydispersity (0.223) and positive zeta potential (+34 mV). The exposure of encapsulated doxorubicin to light (including UV irradiation and daylight) did not provoke any degradation, whereas the nonencapsulated drug was significantly degraded. In vitro studies on keratinocytes (HaCaT) and epidermoid squamous skin carcinoma cells (A-431) disclosed that the encapsulated doxorubicin was more cytotoxic on both cell lines than the pure drug was. More importantly, the cytotoxic concentration of encapsulated doxorubicin in carcinoma cells was approximately two times lower than that in keratinocytes, indicating that it would not affect them. Thus, the loading of doxorubicin into the developed chitosan–albumin nanogel definitely stabilized the drug against photodegradation and increased its antineoplastic effect on the skin cancer cell line.

## 1. Introduction

Nanogels are three-dimensional structures consisting of hydrophilic polymers, which combine the precedence of nanoparticles and hydrogels. They may be formulated through cross-linking via covalent, hydrogen, van der Waals and other interactions. These systems possess various advantages such as biocompatibility and biodegradability, low toxicity and high loading capacity of drugs with different properties. The small diameter of nanogels could enhance the permeability and retention effect (EPR) [1], which is very important for the delivery of anticancer drugs. In addition, the variety of physical and chemical properties of the nanogels provides an opportunity to respond to external factors like pH, ionic strength and temperature. These characteristics make them appropriate as drug delivery systems [2,3,4,5]. It is important to note that nanogels are appropriate as topical drug delivery systems. First, because of their hydrophilicity, they provide efficient moisturizing of the stratum corneum, the main physical and chemical barrier for drugs. Their small size and deformable structure could enhance the trans- and paracellular transport of incorporated drugs, too. Furthermore, local delivery of anticancer drugs through nanogels could minimize their side effects to normal cells by drug delivery in tumor cells. Last but not least, the encapsulation of unstable drugs in nanogels could protect them from internal and external conditions. For instance, the stability of fucoxanthin was improved by its incorporation in a chitosan–glycolipid nanogel [6]. The degradation of curcumin in neutral conditions was significantly decreased through its incorporation in a nanogel based on hyaluronic acid [7].

Natural polymers are preferred as nanogel carriers due to their biocompatibility and nontoxicity. Various scientific groups have prepared nanoscale gels from natural polymers, in particular ovomucin and chito-oligosaccharide [8], hyaluronic acid [9], polyaldehyde dextran and cystamine [10], gelatin [11,12], alginate [13] and chitin [14]. Chitosan is a nontoxic and biodegradable linear polysaccharide which is produced from the chitin in shrimp shells [15]. It represents β-(1→4)-linked D-glucosamine and N-acetyl-D-glucosamine and has free amino groups in its structure. These properties determine its pH-dependent protonation, which enables the formation of polyelectrolyte complexes with oppositely charged polymers and pH-dependent drug release [16]. For instance, a nanogel from chitosan and carboxymethyl–chitosan was prepared, and rifaximin was successfully encapsulated. This system managed to increase the antioxidant effect of the drug and showed improved hemocompatibility [17]. Ashrafi et al. incorporated essential oils from *Mentha piperita* in a chitosan nanogel [18]. The resulting nanogel improved the inhibition of the biofilm produced by *Streptococcus mutants* and inhibited the action of biofilm-related genes. A recent study reported the double loading of glibenclamide and quercetin in a chitosan nanogel with the aim of improving their skin permeation [19]. In vitro safety studies of a glycol chitosan nanogel showed that it did not activate the complement system and did not induce hemolysis, apoptosis, necrosis or cell cycle arrest in the examined cell lines [20]. Another promising natural compound, suitable for preparing nanogels, is albumin. It is a globular protein which is water-soluble and has free carboxyl groups. The main advantages of albumin are its biocompatibility, biodegradability, nontoxicity and the presence of functional groups [21]. Nanogels from bovine serum albumin and acacia gum were obtained, and 5-fluorouracil was successfully encapsulated [22]. The empty nanogels were confirmed to be safe since they did not show significant hemolytic activity and were nontoxic to MCF-7 cells. Furthermore, Liu et al. prepared a nanogel from bovine serum albumin and carboxymethyl cellulose for the codelivery of radionuclide ^131^I and camptothecin. The results demonstrated an increased accumulation in the tumor tissue, absence of hemolysis, great biocompatibility and an enhanced in vivo and in vitro anticancer effect [23]. Albumin could also interact with chitosan, forming a polyelectrolyte complex under specific conditions. For instance, Ay Şenyiğit et al. prepared a nanogel from chitosan, bovine serum albumin and Carbopol 940 via electrostatic interactions and successfully loaded mupirocin [24].

Doxorubicin is one of the most promising antitumor drugs with well-studied cytostatic effects. Along with the well-known activity of doxorubicin against breast cancer, some new studies explored its potential in the treatment of skin cancer [16,25,26,27,28]. Huber et al. developed cationic solid lipid nanoparticles (composed of stearic acid and monoolein) and evaluated their effect in squamous cell carcinoma induced in nude BALB/c mice [28]. The authors reported that the iontophoresis of a referent solution of doxorubicin increased drug penetration in the viable epidermis approximately 4-fold, whereas the iontophoresis of the drug-loaded nanoparticles increased drug penetration approximately 50-fold. The iontophoretic treatment with the nanoparticles led to the inhibition of tumor cell survival and tumor growth in vivo. Tupal et al. reported that doxorubicin encapsulated in solid lipid nanoparticles exerted a superior cytostatic effect in vitro on murine melanoma (B16F10) cells and in vivo on a melanoma-induced model in Balb/C mice [25]. In another study, the encapsulation of doxorubicin in chitosan–alginate nanoparticles provided better accumulation in melanoma cell lines compared to the free drug and maintained its anticancer effect in a syngeneic melanoma mouse model over time [16].

However, one of the drawbacks of doxorubicin is its chemical instability. It was reported that doxorubicin undergoes photolysis and hydrolysis [29,30]. Thus, the drug requires a protective strategy that could ensure its stability during storage and administration. A possible approach to dealing with the low stability of doxorubicin is its encapsulation in nanoparticles. For instance, doxorubicin was protected from UV-induced degradation by embedding it in polyethylene glycol-coated liposomes [31] and silica gels [32]. More recently, a high resistance to UV-induced photolysis was achieved through the encapsulation of doxorubicin into a newly developed nanogel based on pentane-1,2,5-triol and citric acid [33]. Besides stability, drug encapsulation could control the drug delivery as well as improve intracellular transport and therapy effectiveness. Developing a therapeutic system that could combine all these functions would be an appropriate contribution to the current skin cancer treatments.

The aim of the present study was to encapsulate doxorubicin into a nanogel, aiming to improve its chemical stability and to examine the potential of the nanosystem for skin anticancer treatment. Two biopolymers, in particular chitosan and albumin, were selected as nanogel carriers due to their ability to interact in appropriate conditions. The capability of the nanogel to protect doxorubicin against photodegradation as well as the stability of the system itself were evaluated. In addition, the capacity of the nanogel system to enhance the cytostatic activity of doxorubicin in a skin cancer cell line was examined.

## 2. Results and Discussion

### 2.1. Preparation and Characterization of Nanogel

**Figure 1:**
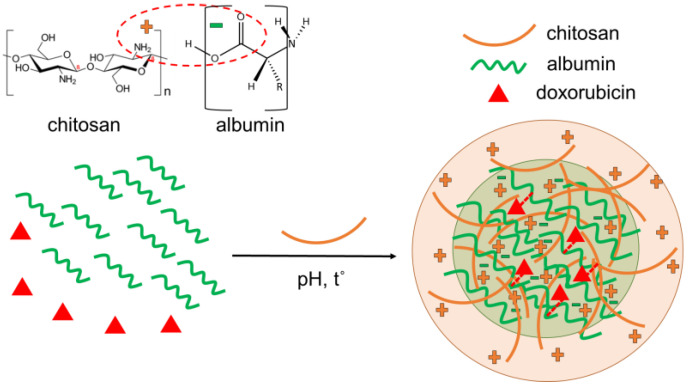
Schematic presentation of the formulation of doxorubicin-loaded chitosan–albumin nanogel (CA-DOX).

**Figure 2:**
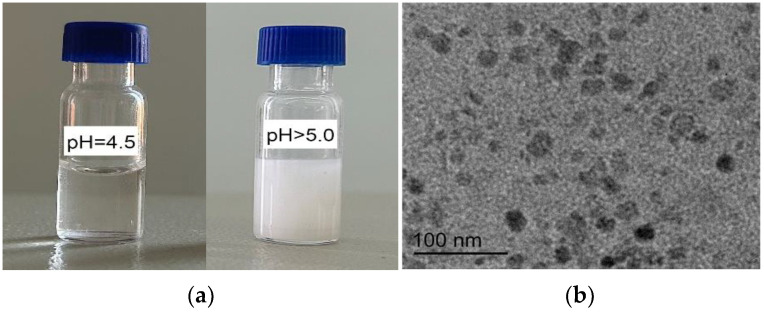
Digital images of empty chitosan–albumin nanogels prepared under heating (78 °C) at different pH values of chitosan–albumin solution (**a**) and transmission electron microscopy of doxorubicin-loaded chitosan–albumin nanogel (**b**).

Chitosan is a linear polysaccharide with functional amino groups, while albumin is a protein with free carboxyl groups. Under specific conditions, in particular pH and temperature, their groups could interact electrostatically, forming a nanogel structure (Figure 1). Yu et al. observed that the electrostatic attractions between chitosan and ovalbumin were enhanced when the pH increased from 4.9 to 5.8, but at higher pH values, precipitates occurred [34]. In the present study, the pH of the mixed solution of chitosan and albumin was varied, falling in the range 4.0–6.0 at a temperature of 78 °C. The results showed that the optimal pH value was 4.54, since reaching a pH value higher than 5.0 (at 78 °C) resulted in a highly unstable dispersion. Figure 2a shows that when the pH was below 5.0, the dispersion was slightly opalescent, while an increase in pH above 5.0 led to turbidity of the nanogel dispersion. It is known that the isoelectric point of bovine serum albumin is 4.5–4.8 [35], indicating that at a pH of 4.54, it will be positively charged. This fact suggested that an electrostatic attraction between chitosan and albumin would have a low contribution to the formation of the nanogel. The heating of the chitosan–albumin solution during the encapsulation was an important condition that enhanced the production of the nanogel due to the gelation of albumin. It was reported that the heating induced a conformational transformation of albumin to a globular form and an intermolecular hydrophobic interaction [34]. Thus, we think that in the present study, the formation of the nanogel was a complex process that involved electrostatic attraction, hydrogen bonding and hydrophobic association between chitosan and albumin chains.

Transmission electron microscopy (TEM) of the optimized system revealed spherical nanoscale particles (Figure 2b). Further, the mean diameter and polydispersity index (PDI) were evaluated through photon correlation spectroscopy. As seen in Table 1, there was no difference between the size of the empty and drug-loaded nanogels. The obtained size was smaller than 200 nm, which would provide selective delivery of the anticancer drug to tumor tissue due to an enhanced permeability and retention [1]. Both polydispersity and zeta potential were similar for empty and drug-loaded nanogels, which showed that the drug incorporation did not influence these parameters (Table 1). The positive charge indicated that the amino groups of chitosan appeared on the surface. This supports the statement of Yu et al. that one part of the chitosan molecules remained as a shell, while the other participated in the formation of the core with the molecules of albumin [34].

The results showed that the encapsulation efficiency and drug-loading degree approximated 75% and 719 µg/mL dispersion, respectively (Table 1). These values were similar to those of other groups [36,37]. According to Li et al. [4], there are three possibilities for incorporating drugs in nanogels, particularly through formulating an outer gel layer and positioning the drug in the inner cavity (1); physical incorporation of drug molecules within the gel network (2); and covalent interactions between the polymers and the drug (3). Here, the most probable mode of drug loading was physical incorporation during formation of the nanogel network.

**Figure 3:**
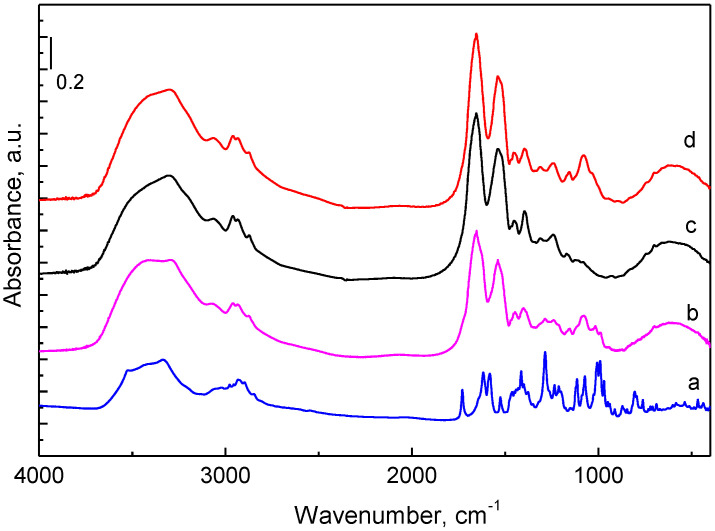
FTIR spectra of doxorubicin (a), doxorubicin-loaded chitosan–albumin nanoparticles (b), albumin (c) and empty chitosan–albumin nanoparticles (d) registered in KBr pellets.

Fourier transform infrared spectroscopy (FTIR) spectra of doxorubicin, albumin, chitosan–albumin nanoparticles and doxorubicin-loaded chitosan–albumin nanoparticles are shown in Figure 3. The bands in the doxorubicin spectrum were in agreement with previously published data [38]. In the FTIR spectrum of albumin, along with the peaks observed for O-H (3300–3550 cm^−1^) and C-H stretching vibrations (2932 cm^−1^ and 2848 cm^−1^), were presented characteristic bands at 1652 cm^−1^ (amide I, C=O stretching), 1536 cm^−1^ (amide II, -NH bending) and 1397 cm^−1^ (amide III, C–N stretching) [39,40]. The spectrum of the chitosan–albumin nanoparticles was close to the albumin spectrum with some additional bands typical for chitosan, particularly at 1078 cm^−1^ and 1022 cm^−1^ due to C-O-C and P=O stretching vibrations [41,42]. The spectrum of doxorubicin-loaded chitosan–albumin nanoparticles (CA-DOX) presented the characteristic bands, which appeared in the spectra of albumin and empty chitosan–albumin nanogel particles. Furthermore, in their spectrum, additional peaks at 1017 cm^−1^ and at 989 cm^−1^ clearly indicated the presence of doxorubicin. This fact confirmed the successful encapsulation of the drug into the chitosan–albumin nanoparticles.

**Figure 4:**
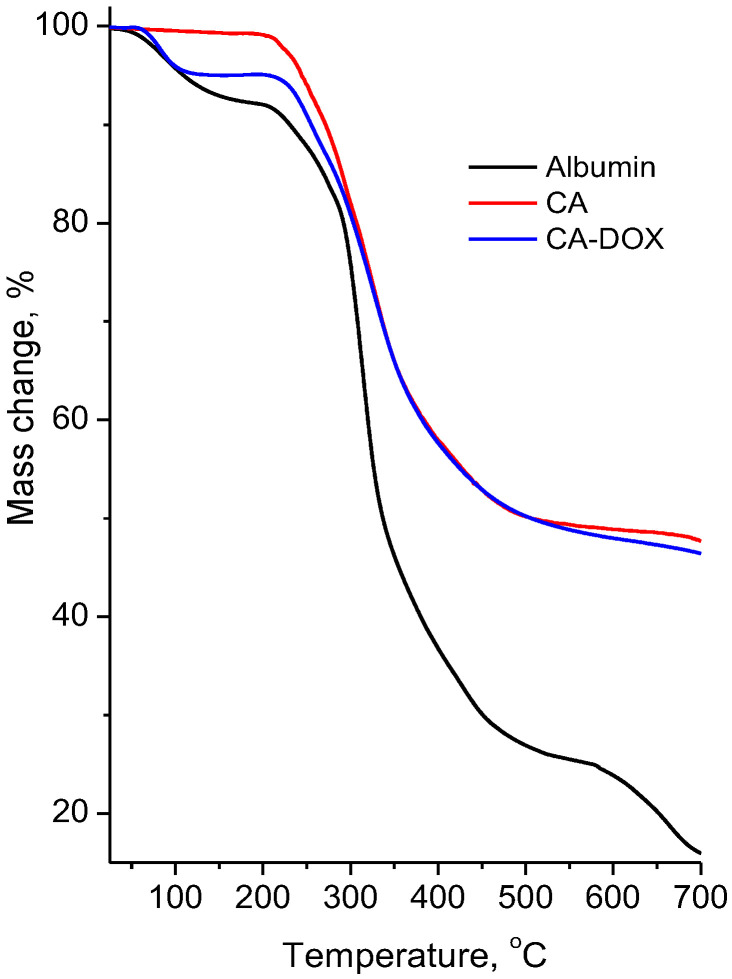
Thermogravimetric curves of albumin, empty chitosan–albumin nanoparticles (CA) and drug-loaded nanoparticles (CA-DOX).

Figure 4 presents the thermogravimetric curves of albumin, empty chitosan–albumin nanoparticles and doxorubicin-loaded chitosan–albumin nanoparticles. In the curve of albumin, three mass-loss steps were observed. The first one of up to ~105 °C was associated with the transition phase (denaturation) with a mass change of 5.5%. The second step occurred at 250–500 °C and was attributed to the decomposition process with a mass loss of 67.5%. The third step at 550–700 °C with a mass loss of 10% could be due to carbonization of the sample. The thermal decomposition curves of the empty chitosan–albumin nanoparticles and doxorubicin-loaded nanoparticles were quite similar in stages and temperatures. They presented two-step thermal degradations. The first one was due to the release of bonded water molecules, the second one to the decomposition of the material. These samples differed only in the mass losses of the first stage—1.5% for the empty chitosan–albumin nanoparticles and 4.5% for the drug-loaded nanoparticles. The second mass losses were 50.8% and 48.5%, respectively. Thus, the addition of chitosan to albumin led to the thermal stabilization of the mixed carrier, which remained even when the drug was loaded.

**Figure 5:**
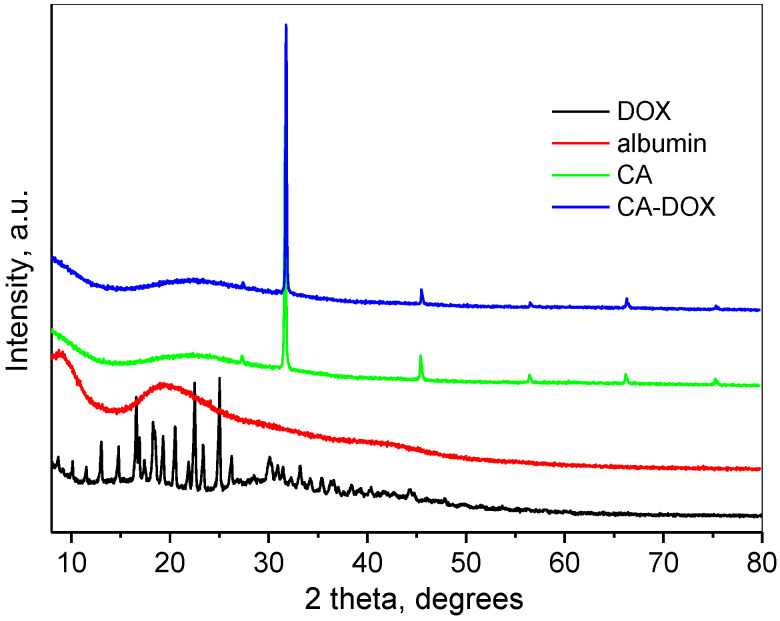
XRD pattern of doxorubicin (DOX), albumin, empty chitosan–albumin nanoparticles (CA) and the drug-loaded nanoparticles (CA-DOX).

The powder X-ray diffraction (XRD) pattern of doxorubicin consisted of a large number of sharp peaks at 2θ angles below 50° 2θ, indicating its crystalline nature (Figure 5). The pattern was close to already published data for doxorubicin nitrate [43]. The XRD pattern of albumin represented amorphous material with broad peaks at about 10° and 20° 2θ. The powder diffraction pattern of empty chitosan–albumin nanogel particles and drug-loaded nanoparticles consisted of an amorphous broad peak, slightly shifted towards higher 2θ degrees in comparison to those of pure albumin. Some sharp peaks of NaCl due to the preparation procedure were registered in both diffraction patterns. The disappearance of the sharp peaks of doxorubicin along with the clear presence of its characteristic peaks in the FTIR spectrum indicated that the drug was encapsulated in the chitosan–albumin nanoparticles.

**Figure 6:**
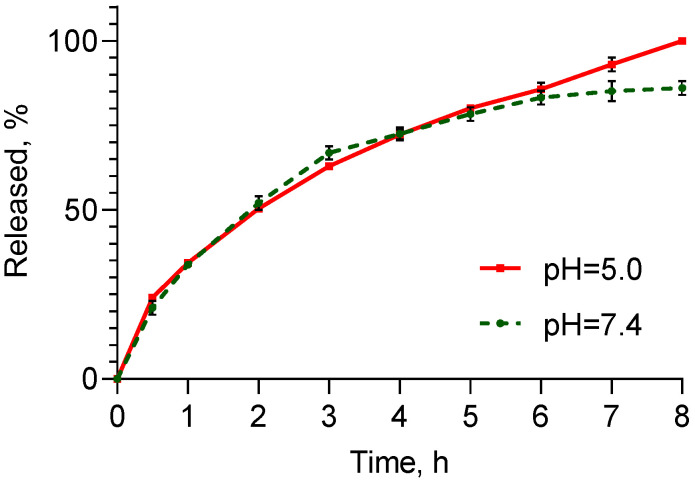
In vitro drug release profiles of doxorubicin-loaded nanogel in media with pH value of 5.0 and 7.4 (*n* = 3).

The in vitro release tests were conducted in media with pH values of 5.0 and 7.4, representing the pH range of skin layers, particularly from the slightly acidic pH of the stratum corneum to neutral values in the vital layers, respectively (Figure 6). In general, the release process was faster compared to previously reported data for drug release from similar nanogels. For example, Ay Şenyiğit et al. observed a slower release of mupirocin from chitosan–albumin nanoparticles that was completed after approximately 24 h [24]. In the present study, the faster release could be explained by the more hydrophilic properties of doxorubicin. As shown, there was a slight difference between the release rate in both media, being faster in the acidic medium after 6 h of the process. It is well-known that the solubility of doxorubicin in an acidic medium (1.04 mg/mL at pH = 5.0) is higher than that in an alkaline medium (0.51 mg/mL at pH = 7.4) [44]. Thus, it seems that another factor, particularly the different degree of protonation of chitosan in both media, had a greater influence on the process. Chitosan is protonated at pH = 5.0, while at pH = 7.4 it is uncharged and insoluble. In our opinion, because of low chitosan solubility at the higher pH, the outer chitosan nanogel layer probably shrinks, and this phenomenon could slow down the release. To evaluate the mechanism of drug release, zero-order, first-order and diffusion-controlled release models were used to analyze the data. In the acid buffer, the greatest correlation coefficient (r^2^) was found for the Higuchi model, indicating that the release process was controlled by diffusion through the nanogel structure (Table 2). In the alkaline medium, the correlation coefficient for the first order was higher than that for the Higuchi model. As seen in Figure 6, after 6 h, the drug release in this buffer diminished, meaning that the rate depended on the remaining drug concentration. This observation was in agreement with the slightly higher correlation coefficient found for the first order.

### 2.2. Stability of Doxorubicin and Nanogel Formulation

**Figure 7:**
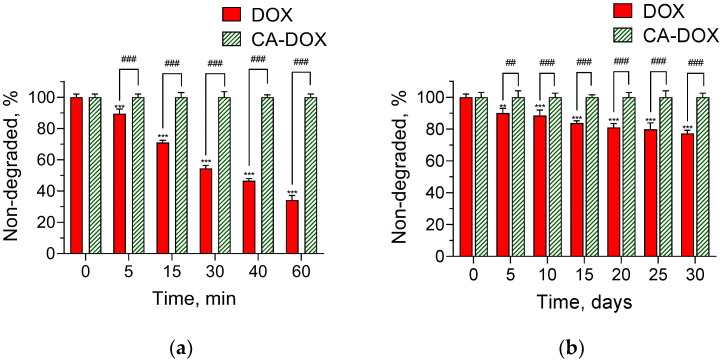
Photolysis of pure doxorubicin (DOX) and encapsulated doxorubicin (CA-DOX) under UV irradiation (**a**) and daylight exposure (**b**); ** *p* < 0.01; *** *p* < 0.001 vs. DOX (0 min/0 days) group (one-way ANOVA with Dunnett post-test); ^##^ *p* < 0.01; ^###^ *p* < 0.001 between groups (two-way ANOVA and Holm–Šidák multiple comparisons test).

When dissolved in water (e.g., in an injection solution) doxorubicin undergoes photolytic and hydrolytic degradation. This leads to its low stability and may be an obstacle for its practical application. Nowadays, the formulation of the drug in appropriate drug delivery systems can be used to avoid the formation of degradation derivatives upon irradiation or sunlight exposure [33]. The presumption of the present study was that the inclusion of doxorubicin in nanogel could have a protective effect against light degradation. Thus, the nanogel and a referent solution were treated with UV irradiation, and the concentration of nondegraded doxorubicin in both samples was evaluated over certain intervals of time (Figure 7). High-performance liquid chromatography (HPLC) revealed a very well-pronounced decrease in the concentration of doxorubicin in the aqueous solution with time. In contrast, the UV irradiation did not provoke degradation of doxorubicin encapsulated in the nanogel, where there was no decrease in the concentration even after 60 min of the treatment (Figure 7a). In particular, the degradation of doxorubicin in the referent solution was 10% at the first 5 min of irradiation and reached 66% at the first hour. For comparison, after 60 min of irradiation, degradation of doxorubicin in the nanogel was not registered. Similarly, the formulation of doxorubicin in a cross-linked nanogel based on citric acid and pentane-1,2,5-triol prevented doxorubicin photolysis under UV irradiation [33]. In particular, approximately 90% nondegraded doxorubicin was registered after 60 min irradiation of the nanogel particles. Further, stability studies were performed after exposure of the solution and the developed nanogel to daylight. The results showed a similar pattern to that obtained under UV irradiation (Figure 7b). In particular, 10% of the drug in the referent solution degraded on the fifth day, whereas for the encapsulated doxorubicin, such a decrease was not observed. After 30 days of exposure, the nondegraded doxorubicin in the referent solution dropped down to a level of 77%, whereas the encapsulated doxorubicin maintained its stability (Figure 7b). These data revealed that the loading of doxorubicin into the nanogel definitely stabilized the drug against photodegradation. The statistical significance between the nonencapsulated and encapsulated groups confirms that. The protection could be explained by the inner location of doxorubicin in the nanogel structure. Thus, the studies revealed the protective capability of the chitosan–albumin nanogels and affirmed the practical importance of the obtained drug delivery system.

**Figure 8:**
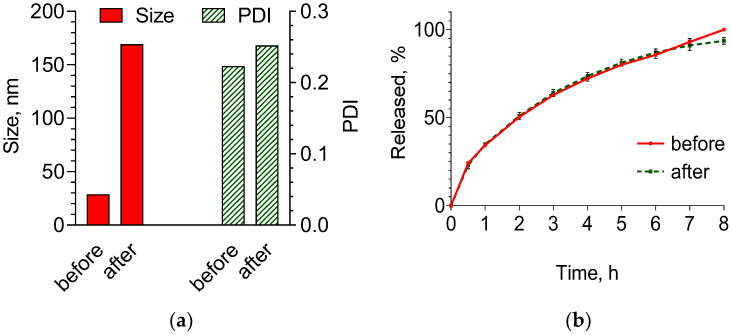
Average diameter and polydispersity index (PDI) of doxorubicin-loaded nanogel particles before and after lyophilization (**a**) and release profiles from the nanoparticles before and after lyophilization (**b**).

Lyophilization is one of the most useful methods for processing nanoparticle dispersions in anhydrous form with high storage stability [45,46]. However, the lyophilization could affect the physicochemical properties and release characteristics of nanoparticles. In the present study, the stability of the formulated nanogel system after lyophilization was evaluated considering changes in the mean diameter, polydispersity and in vitro release rate (Figure 8a,b). The comparison of the mean diameter of the nanogel before and after lyophilization showed an increase in the size. The mean diameter of the nanogel increased to 170 nm, as shown in Figure 8a. The reason for such augmentation could be the aggregation of particles during the freezing step, since their concentration increases during that time [47]. However, the diameter dimension was still in the range of the optimal size regarding the well-known EPR theory [1]. The polydispersity of the nanogel particles after lyophilization was low (0.28), suggesting a narrow size distribution and safe application. Some studies reported an increase in the release rate after lyophilization due to the formation of pores in the nanoparticles [47]. The release profiles in the acidic medium before and after lyophilization (Figure 8b) were compared by calculation of similarity factor. The calculation showed that the value of the similarity factor was higher than 50 (f_2_ = 91.85), indicating that both profiles were similar. Thus, the lyophilization did not influence the nanogel structure and it remained stable.

### 2.3. Cytotoxicity Studies

**Figure 9:**
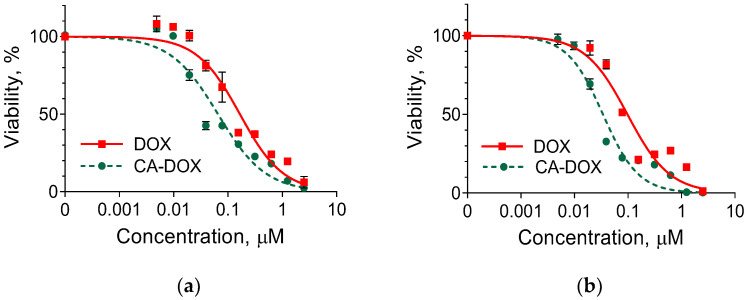
Viability of keratinocytes HaCaT (**a**) and epidermoid squamous skin carcinoma cells A-431 (**b**) after 72 h treatment with doxorubicin solution (DOX) and encapsulated in nanogel doxorubicin (CA-DOX).

Doxorubicin could be applied in skin cancer treatment, and the effect of the free and encapsulated drug has been proved in vitro and in vivo, in particular in melanoma cell lines (B16-F10 and B16-OVA) and a melanoma mouse model [16,25], in human melanoma cells (A375) [26,27] and in nude BALB/c mice with squamous cell carcinoma [28]. All authors confirmed that incorporating doxorubicin in nanoparticles could improve its effect and application. In our study, we evaluated the cytotoxic effect of the pure drug and the encapsulated drug on immortalized keratinocytes (HaCaT cell line) and nonmelanoma epidermoid squamous skin carcinoma (A-431 cell line) (Figure 9a,b). The results showed dose-dependent cytotoxicity of the pure and the encapsulated drug on both cell lines. As seen in Table 3, there was an almost 2.5-fold decrease in the IC_50_ of encapsulated doxorubicin on the healthy keratinocytes and about 2.9-fold on the cancerous cells in comparison with the pure drug. These results showed that the nanogel system managed to enhance the cytotoxic effect of doxorubicin in both cell lines. However, there was also an approximately 1.9-fold difference in the IC_50_ values of encapsulated doxorubicin between the HaCaT and A-431 cells in favor of the healthy cells. The statistical difference between the effects of encapsulated doxorubicin on normal keratinocytes and epidermoid carcinoma cells is presented in Table 4. This difference was significantly pronounced in the concentration range between 0.039 and 1.25 µM in which the IC_50_ values were determined. In addition, the cytotoxic concentrations of doxorubicin in epidermoid carcinoma cells were not high enough to affect the keratinocytes. This confirmed the precedence of the doxorubicin-loaded nanogel, namely, the higher cytotoxicity on the carcinoma cells.

## 3. Materials and Methods

### 3.1. Materials

Doxorubicin hydrochloride, chitosan (Mv 110,000–150,000), bovine serum albumin (fraction V), fetal bovine serum, acetonitrile (HPLC grade) and orthophosphoric acid (HPLC grade) were purchased from Sigma Chemical Co. (Merck, Darmstadt, Germany). For the in vitro cytotoxicity studies, keratinocytes HaCaT (CLS Cell Lines Service, 300493, Eppelheim, Germany) and epidermoid squamous skin carcinoma cells A-431 (CLS Cell Lines Service, 300112, Eppelheim, Germany) were used.

### 3.2. Preparation of Empty Nanoparticles and Doxorubicin-Loaded Nanogel

Chitosan–albumin nanogel was produced after slight modification of previously reported procedures [34,48]. The ratio between chitosan and bovine serum albumin in the present study was 1:5 (*wt*/*wt*). Briefly, 1 mL of 0.5% chitosan solution in buffer (pH 1.2) was added to 5 mL of 0.5% aqueous solution of albumin. Then the mixture was stirred at 700 rpm for 90 min. The necessary amount of 1 M NaOH was dropped until the pH value reached 4.54 and the mixture was heated at 78 °C for 20 min. Then, the nanogel was stirred for 3 h (700 rpm) in order to avoid obtaining large particles. For the preparation of drug-loaded nanogel particles, an aqueous solution of doxorubicin (3 mg/3 mL) was added to the aqueous solution of albumin following all other stages of the procedure. Then the loaded nanogel dispersion was filtered (0.2 µM), and the nonencapsulated doxorubicin in the rinsing aqueous filter fraction was determined via HPLC method (Thermo Scientific UltiMate Dionex 3000 SD, Chromeleon 7.2 SR3 Systems, Thermo Fisher Scientific, Waltham, MA, USA). The system consisted of a Phenomenex C18 Column—Luna (250 mm × 4.60 mm, particle size 5 μm), an isocratic mobile phase containing acetonitrile, water and orthophosphoric acid (volume ratio 30:70:0.2, respectively) and a flow rate of 1.0 mL/min. The detection wavelength was set at 254 nm. The column and the HPLC system were kept at temperature 25 °C ± 1 °C.

The encapsulation efficiency (EE) and loading degree (LD) were calculated by applying the following equations:

EE = (Total amount of drug − Non-loaded drug)/Total amount of drug,(1)

LD = (Total amount of drug − Non-loaded drug)/Volume of drug-loaded nanogel dispersion(2)

### 3.3. Characterization of the Nanogel

Transmission electron microscopy was conducted using an HR STEM JEOL JEM 2100 (Tokyo, Japan). Photon correlation spectroscopy at a scattering angle of 90° was applied in order to determine the diameter and polydispersity of the nanogel particles (Zetasizer NanoBrook 90Plus PALS, Brookhaven Instruments Corporation, Holtsville, NY, USA). Zeta potential was evaluated via the phase analysis light scattering (PALS) method at a scattering angle of 15°. The nanogel dispersions were diluted 10 times, placed in polystyrene cuvettes (BRAND GMNB, Wenheim, Germany) and measured under the scattering angle described above.

IR spectra were collected with a Nicolet iS5 FTIR spectrometer (Thermo Fisher Scientific, Waltham, MA, USA) accumulating 64 scans at spectral resolution of 2 cm^−1^.

Thermogravimetric analyses were performed in dynamic conditions using LABSYSEvo, SETARAM (Caluire-et-Cuire, France) in Ar within a temperature range of 25–700 °C with a heating rate of 10 °C/min.

Powder X-ray diffraction patterns of doxorubicin, albumin, chitosan–albumin and loaded nanoparticles were recorded in the 5–80° 2θ range with a step of 0.02° with a Bruker D8 Advance diffractometer (Bruker Corporation, Billerica, MA, USA) with Cu Kα radiation and a LynxEye detector.

### 3.4. In Vitro Release of Doxorubicin from the Nanogel

In vitro release studies were performed via dialysis method in a shaking water bath maintaining 32 °C temperature (IKA Labortechnik HS-B20, Staufen, Germany) [49] as release media buffers having pH values of 5.0 and 7.4 were applied. Thus, 2.5 mL of the nanogel dispersion was introduced in a dialysis membrane (10,000 MWCO, Spectrum Labs, San Francisco, CA, USA) and placed in 60 mL of the buffer. Samples of 5 mL were taken from the external medium every hour, and the same amount of fresh medium was returned to maintain sink conditions. The concentration of released doxorubicin was determined as described above.

The drug release kinetics were evaluated via fitting the data to zero-order (Equation (3)), first-order (Equation (4)) and Higuchi (Equation (5)) models:

C_t_ = C_0_ + K_0_·t (3)

where C_t_ represents the amount of active agent released during the time t; C_0_ is the initial concentration of the drug released; and K_0_ is the zero-order rate constant.

ln(C_i_ − C_t_) = ln(C_i_) − K_1_·t (4)

where C_t_ represents the amount of active agent released during the time t; C_i_ is the initial concentration of the drug before release; and K_1_ is the first-order rate constant.

C_t_ = K_H_·t^1/2^ (5)

where C_t_ is the amount of drug released during the time t and K_H_ is the release constant of Higuchi.

### 3.5. Stability Studies of Doxorubicin

Photostability of pure and encapsulated doxorubicin was evaluated by applying UV irradiation (Dymax 5000-EC UV equipment with a 400 W metal halide flood lamp) at a dose rate of 5.7 J/cm^2^·min. The dispersion of the nanogel and aqueous solution of doxorubicin (at the same concentration) were placed in glass vials, and the concentration of doxorubicin in both samples was determined at defined intervals as described above. Furthermore, the changes in the stability of the drug were evaluated also under storage in daylight conditions.

The stability of the nanogel formulation after lyophilization was examined by measurements of mean diameter, polydispersity and in vitro drug release.

### 3.6. In Vitro Cytotoxicity Studies

In vitro cytotoxicity studies of pure and encapsulated doxorubicin were conducted on normal keratinocytes HaCaT and epidermoid squamous skin carcinoma cells A-431. The cell lines were maintained in culture medium DMEM (DMEM-HPA, Capricorn^®^, Ebsdorfergrund, Germany) supplemented with 10% fetal bovine serum and 4 mM L-glutamine. The cell lines were incubated under standard conditions (5% CO_2_, 37 °C, maximal humidity) and subcultured every 4th day by splitting 1:8 after washing with phosphate buffered saline (PBS, pH 7.4) (TS1101, HiMedia, Mumbai, India) and cell detachment with Accutase^®^ (ACC-1B, Capricorn^®^, Germany).

The cell viability assay was performed according to ISO 10993-5-2009 [50], Annex C, which is based on the MTT reduction assay [51]. Briefly, cells were seeded in 96-well plates in a starting density of 0.7 × 10^5^ cells/mL and incubated for 24 h under sterile conditions (Laminar Air Flow Telstar Bio II Advance, Barcelona, Spain) until entering the log phase of the growth curve. Thereafter, they were exposed for 72 h to pure or encapsulated doxorubicin in concentrations ranging from 0.005 up to 2.5 µM in twofold serial dilutions. All experiments were performed in triplicate, wherein every sample was repeated four times. Cell viability was determined with the MTT dye in final concentration of 0.05 mg/mL after 2 h of incubation at 37 °C. The culture medium was removed and the formazan crystals formed were dissolved in isopropyl alcohol containing 5% HCOOH (Chimspektar OOD, Sofia, Bulgaria). The absorbance was measured at λ = 550 nm (reference filter 690 nm) against a blank solution (the organic solvent) using an Absorbance Microplate Reader EL-800 (Bio-Tek Instruments Inc., Winooski, VT, USA).

### 3.7. Statistical Analysis

The results from the cell viability test were statistically analyzed using GraphPadPrism, version 6.01 for Windows (GraphPad Software, San Diego, CA, USA). One-way ANOVA with Dunnett’s multiple comparison post-test and two-way ANOVA followed by Holm–Šidák test was performed. The median inhibitory concentrations (IC_50_) were calculated with the “log(inhibitor) vs. normalized response—Variable slope” model based on the equation Y = 100/(1 + 10^((LogIC50-X)*HillSlope)), wherein each concentration was repeated in quadruplicate.

## 4. Conclusions

A nanogel from the natural polymers chitosan and albumin was successfully obtained, and doxorubicin was incorporated into the nanogel, reaching sufficient encapsulation efficiency. The developed nanosystem improved the stability of doxorubicin by decreasing its photosensitivity, which is a problematic issue for this drug. This study revealed the cytotoxic activity of doxorubicin on a selected skin carcinoma cell line. Furthermore, by loading doxorubicin into the nanogel, its antineoplastic effect on skin cancer cells was enhanced, which confirmed the precedence of the developed drug delivery system.

## Figures and Tables

**Table 1:** Average diameter, polydispersity index (PDI) and zeta potential of empty chitosan–albumin nanoparticles (CA) and doxorubicin-loaded chitosan–albumin nanoparticles (CA-DOX), encapsulation efficiency (EE) and loading degree (LD) of doxorubicin.

Nanoparticles	Average Diameter, nm	PDI	Zeta Potential, mV	EE, %	LD, µg/mL
CA	29.2 ± 5	0.241	+35.5	-	-
CA-DOX	28.8 ± 6	0.223	+34.4	74.7	718.7

**Table 2:** Correlation coefficients (r^2^) for zero-order, first-order and Higuchi models calculated through kinetic analysis of in vitro release data.

pH of the Medium	Zero Order	First Order	Higuchi Model
pH 5.0	0.9616	0.9751	0.9984
pH 7.4	0.8612	0.9681	0.9530

**Table 3:** IC_50_ of doxorubicin in aqueous solution (DOX) and encapsulated in nanogel (CA-DOX) in HaCaT and A-431 cell lines after 72 h treatment.

Cell Line	DOX		CA-DOX	
	IC_50_, µM	95% CI	IC_50_, µM	95% CI
HaCaT	0.166	0.138–0.199	0.066	0.053–0.081
A-431	0.098	0.080–0.119	0.034	0.029–0.041

**Table 4:** Comparative analysis of the differences in the cytotoxic activity of encapsulated doxorubicin (CA-DOX) on HaCaT and A-431 cells (two-way ANOVA and Holm–Šidák multiple comparisons test).

Concentrations (µM) of CA-DOX, HaCaT vs. A-431	Mean 1	Mean 2	Mean Difference	95% CI of Difference	Significance	Adjusted *p* Value
0.004883 vs. 0.004883	105.1	97.76	7.372	3.085 to 11.66	****	<0.0001
0.009766 vs. 0.009766	100.4	93.67	6.780	2.494 to 11.07	***	0.0002
0.019531 vs. 0.019531	75.22	69.37	5.849	1.180 to 10.52	**	0.0059
0.039063 vs. 0.039063	41.88	32.72	9.161	4.874 to 13.45	****	<0.0001
0.078125 vs. 0.078125	42.60	22.45	20.15	15.86 to 24.44	****	<0.0001
0.156250 vs. 0.156250	30.68	20.80	9.879	5.593 to 14.17	****	<0.0001
0.312500 vs. 0.312500	22.71	18.04	4.670	0.384 to 8.96	*	0.0190
0.625000 vs. 0.625000	18.18	11.34	6.840	2.553 to 11.13	***	0.0002
1.250000 vs. 1.250000	7.012	0.5607	6.451	2.164 to 10.74	***	0.0004
2.500000 vs. 2.500000	2.477	0.3901	2.087	−2.200 to 6.37	ns	0.8175

Legend: CI—confidence interval; ns—not significant; *—significant for *p* < 0.05; **—significant for *p* < 0.01; ***—significant for *p* < 0.001; ****—significant for *p* < 0.0001.

## Data Availability

Data is contained within the article.
